# Book Reviews

**Published:** 1874-05-15

**Authors:** 


### Book Reviews.

ANNOUNCEMENT.—At the request of Dr. H. von Ziemssen, Professor of Clinical Medicine at Erlangen, a number of the most eminent clinical instructors of Germany have undertaken to prepare, in a series of independent treatises, a complete *Encyclopædia of the Practice of Medicine;* the incentive to this labor is the great need which has been felt the past year or two of a work, which fully corresponded to the present standpoint of clinical medicine. This Encyclopædia will embrace the entire range of Special Pathology and Therapeutics, and will be completed in fifteen volumes, large octavo, of from 500 to 700 pages each. The list of contents of each volume, gives the names of the authors and the special departments which they have undertaken. While the work of each writer will bear the stamp of individuality, there will be an effort made to give to each subject the prominence and space due to it only—that the harmony of the entire work may be preserved. It is designed that the Encyclopædia shall be, par excellence, a Practical Handbook for Physicians; and for this reason especial attention has been given to clear and systematic arrangement.

For the value of the whole work, as well as the separate departments, the names of the writers are a sufficient guarantee. Each volume will have a full and carefully prepared index.

Messrs. Wm. Wood & Co. announce that they will publish by *Subscription* a translation of this work. The translating will be done by professional gentlemen, many of them former students of the writers of the different treatises, under the supervision of a responsible chief. Great care will be taken with the mechanical execution of the volume. The type will be large and clear, the paper fine, and the engravings electrotypes of the originals. It is proposed to publish three to four volumes a year, at, as nearly as possible, regular intervals, in order to distribute the cost of subscription equally over about four years.

Terms of subscription, payable upon the delivery of each volume: Fifteen volumes, octavo, muslin binding, per vol., $5.00; fifteen volumes, octavo, leather binding, per volume, $6.00; fifteen volumes, octavo, half morocco binding, per vol., $7.50.

A circular, giving the names of the authors, and the table of contents of each volume, can be had on application to the publishers.

---

CHICAGO JOURNAL OF NERVOUS AND MENTAL DISEASE.—The second number of this valuable new journal has been received. It is considerably larger than the first number, containing 156 pages. The original contents includes the second lecture on the "Pathology of the Vaso-Motor Nervous System," by J. S. Jewell, M. D.; "Mechanism of Reflex Nervous Action in Normal Respiration," by Prof. Austin Flint, Jr., M.D., with Remarks by Prof. J. C. Dalton, M.D.; "Speech as a Reflex Act—The Phono-Motor Nervous Centre," by Dr. E. Onimus; "Some Remarks on the Theory of Inhibitory or Reflex Paralysis," by C. Handfield Jones, M.B., F.R.S.; "Notes of some Recent Cases of Deafness, following Cerebro-Spinal Meningitis," by Samuel J. Jones, A.M., M.D.; "A Case of Chorea—A New Method of Treatment Suggested," by Prof. Ransom Dexter, M.D., and "Nervous Sore Throat." The Periscope contains a full, complete summary of progress in the study of the Anatomy and Physiology, the Pathology, and the Therapeutics of the Nervous System—gleaned from a wide field of home and foreign journals.

---

ARCHIVES OF ELECTROLOGY AND NEUROLOGY.—The Contents of the first No. of this semi-annual journal is as follows: "Electrolysis and Croton Chloral," by Julius Althaus, M.D., of London; "Case of Complete Paralysis of one Recurrent Laryngeal Nerve," by F. I. Knight, M. D., of Boston; "Cataleptiform Paraplegia," by Prof. Moritz Benedict, M. D., of Vienna; "The Nature of Electricity," by Prof. Henry T. Eddy, of Princeton College; "Electrolysis in the Treatment of Strictures of the Urethra," by Robert Newman, M.D., of New York; "A Case of Hysterical Hemiplegia," by Prof. J. L. Cabell, M.D., University of Virginia; "Tinnitus Aurium treated by the Galvanic Current," by T. F. Rumbold, M. D., of St. Louis; "Hysteria and Spinal Irritation treated by Central Galvanization," by W. F. Hutchinson, M.D., of Providence, R. I.; "Some Recent Investigations into the Functions of the Human Brain," by Prof. Roberts Bartholow, M.D., of Cincinnati; "A New Method of Treating Malignant Tumors by Electrolyzing the Base," by George M. Beard, M.D.; "Case of Scirrhus of the Rectum treated by Electrolysis, with Remarks," by Prof. A. B. Crosby, M. D., of New York; "Experimental Researches in the Physiology of the Brain," by George M. Beard, M. D.; "Complete Hemiplegia followed by Rapid Recovery—Probable Embolus," by Prof. Samuel G. Armor, M.D., of Brooklyn; "Medical Jurisprudence and the Medico-Legal Society of New York," by Clark Bell, Esq., of New York; "The Cabinet Battery," by Geo. M. Beard, M.D.; Gleanings from Foreign Journals; Reviews of Althaus, Byrne, and Arthius; New York Society of Neurology and Electrology; The New York Neurological Society, etc.

---

THE AMERICAN JOURNAL OF OBSTETRICS.—With the present number of this journal, which commences the seventh volume, Dr. B. F. Dawson, by whom it has been so ably edited since its foundation, retires from its management. Dr. Paul F. Munde succeeds to the editorial charge.

As now issued by Messrs. Wm. Wood & Co., the journal has been increased in size so as to give about thirty pages additional reading matter.

Dr. Dawson, in his farewell editorial, states that, "although no longer connected with the *Journal*, I shall watch its future with paternal interest, and it will always be my pleasure to do whatever may help to increase its usefulness and furlher its interests." To this end Dr. Dawson offers an annual prize of $150, gold, for the best essay on some subject to be announced at the beginning of each year. The subject for the present year, as announced, is *Congenital Deformities, and Diseases depending on Maladies of the Uterus or Membranes.* The competing essays to be sent to the publishers, Wm. Wood & Co., on or before April 15th, 1875.

